# Supplementary material for: Fingolimod Prevents Neuroinflammation but Has a Limited Effect on the Development of Ataxia in a Mouse Model for SCA1
Source: Int J Mol Sci. 2025 May 14;26(10):4698. doi: 10.3390/ijms26104698 (PMC12111356; doi:10.3390/ijms26104698)
Supplement: Supplementary file 1 [file ijms-26-04698-s001.zip › Suppl. Fig. 1.pdf]

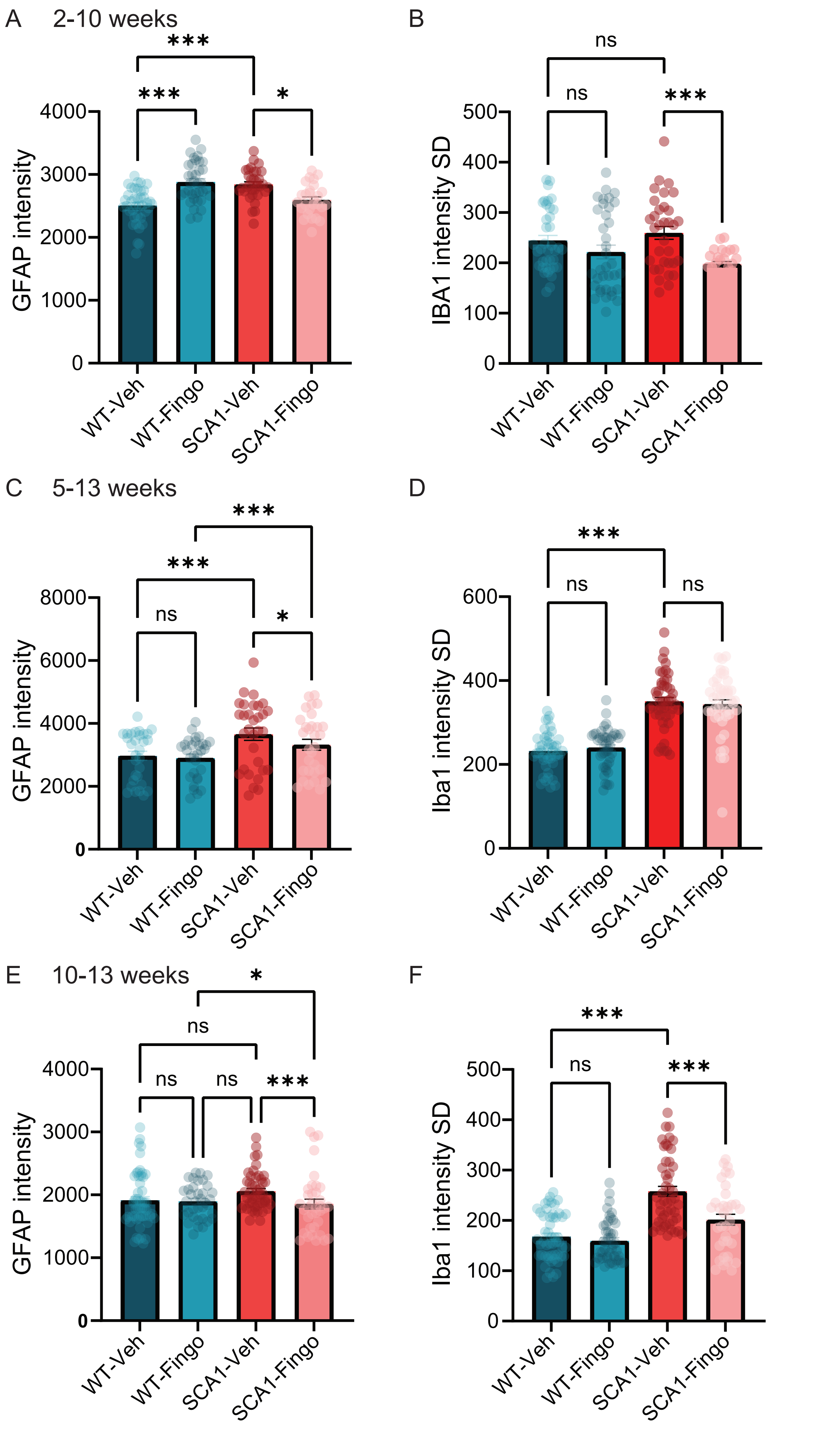

Supplementary Figure 1. GFAP expression intensity and and Iba1 standard deviation of intensity for the entire cerebellum of mice treated during week 2-10 (A-B), 5-13 (C-D) and 10-13 (E-F). Related to Figures 2, 5 and 8.
