# Supplementary material for: Fingolimod Prevents Neuroinflammation but Has a Limited Effect on the Development of Ataxia in a Mouse Model for SCA1
Source: Int J Mol Sci. 2025 May 14;26(10):4698. doi: 10.3390/ijms26104698 (PMC12111356; doi:10.3390/ijms26104698)
Supplement: Supplementary file 1 [file ijms-26-04698-s001.zip › Suppl. Fig. 4-change.pdf]

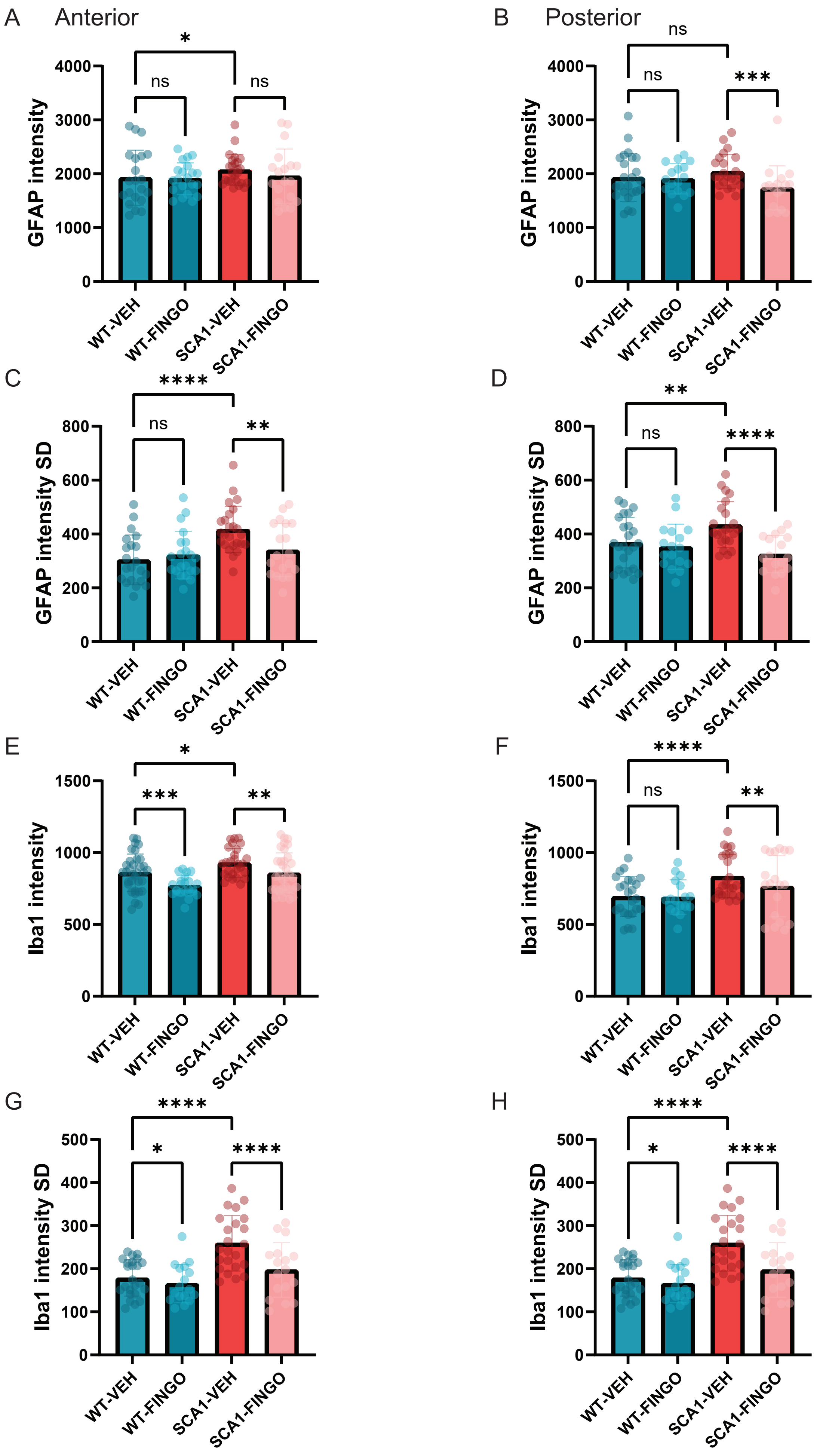

Supplementary Figure 4. GFAP and Iba1 expression intensity and standard deviation of intensity for the anterior and posterior cerebellum of mice treated during week 10-13. Related to Figure 8
