# Supplementary material for: Fingolimod Prevents Neuroinflammation but Has a Limited Effect on the Development of Ataxia in a Mouse Model for SCA1
Source: Int J Mol Sci. 2025 May 14;26(10):4698. doi: 10.3390/ijms26104698 (PMC12111356; doi:10.3390/ijms26104698)
Supplement: Supplementary file 1 [file ijms-26-04698-s001.zip › supplementary table.pdf]

2-10 Rotarod data – mix effects model (REML) WT-VEH vs SAC1-VEH

Uncorrected Fisher's LSD

Week	Predicted (LS) mean diff.	95.00% CI of diff.	Below threshold?	Summary	Individual P Value
5	-34.53	-70.72 to 1.648	No	ns	0.0609
6	-34.91	-71.09 to 1.271	No	ns	0.0582
7	-40.70	-76.88 to -4.516	Yes	*	0.0284
8	-58.65	-94.83 to -22.46	Yes	**	0.0021
9	-59.25	-95.43 to -23.07	Yes	**	0.0020
10	-71.19	-107.4 to -35.01	Yes	***	0.0003

2-10 Rotarod data – mix effects model (REML) SCA1-VEH vs SAC1-FINGO

Uncorrected Fisher's LSD

Week	Predicted (LS) mean diff.	95.00% CI of diff.	Below threshold?	Summary	Individual P Value
5	32.72	-24.46 to 89.91	No	ns	0.2547
6	7.608	-49.58 to 64.79	No	ns	0.7896
7	-7.35	-64.54 to 49.83	No	ns	0.7966
8	29.36	-27.83 to 86.54	No	ns	0.3061
9	-5.925	-63.11 to 51.26	No	ns	0.8354
10	14.12	-43.07 to 71.30	No	ns	0.621

2-10 12mm Balance Beam time pried – mix effects model (REML)) WT-VEH vs SAC1-VEH

Uncorrected Fisher's LSD

Week	Predicted (LS) mean diff.	95.00% CI of diff.	Below threshold?	Summary	Individual P Value
4	-0.3150	-1.608 to 0.9777	No	ns	0.6266
5	-0.3344	-1.627 to 0.9583	No	ns	0.6055
6	1.107	-0.1857 to 2.400	No	ns	0.0916
7	1.936	0.6436 to 3.229	Yes	**	0.0041
8	1.845	0.5526 to 3.138	Yes	**	0.0061
9	2.097	0.8042 to 3.390	Yes	**	0.0020
10	2.662	1.370 to 3.955	Yes	***	0.0001

2-10 12mm Balance Beam time pried – mix effects model (REML) SCA1-VEH vs SAC1-FINGO

Uncorrected Fisher's LSD

Week	Predicted (LS) mean diff.	95.00% CI of diff.	Below threshold?	Summary	Individual P Value
4	1.433	-1.933 to 4.800	No	ns	0.3476
5	1.033	-0.6965 to 2.763	No	ns	0.2007
6	0.3583	-1.604 to 2.321	No	ns	0.6789
7	-0.1250	-1.888 to 1.638	No	ns	0.8716
8	1.433	-0.6549 to 3.522	No	ns	0.1486
9	1.117	-0.8513 to 3.085	No	ns	0.2216
10	-0.2000	-1.798 to 1.398	No	ns	0.7759

2-10 12mm Balance Beam foot slips – mix effects model (REML) WT-VEH vs SAC1-VEH

Uncorrected Fisher's LSD

Week	Predicted (LS) mean diff.	95.00% CI of diff.	Below threshold?	Summary	Individual P Value
4	-0.1034	-1.261 to 1.054	No	ns	0.8584
5	0.4622	-0.6955 to 1.620	No	ns	0.4263
6	0.5695	-0.5883 to 1.727	No	ns	0.3278
7	2.188	1.031 to 3.346	Yes	***	0.0004
8	2.036	0.8778 to 3.193	Yes	***	0.0009
9	2.177	1.019 to 3.334	Yes	***	0.0004
10	2.258	1.100 to 3.416	Yes	***	0.0003

2-10 12mm Balance Beam foot slips – mix effects model (REML) SCA1-VEH vs SAC1-FINGO

Uncorrected Fisher's LSD

Week	Predicted (LS) mean diff.	95.00% CI of diff.	Below threshold?	Summary	Individual P Value
4	0.2000	-1.539 to 1.939	No	ns	0.8182
5	0.3333	-1.406 to 2.073	No	ns	0.7018
6	-0.1750	-1.914 to 1.564	No	ns	0.8406
7	-0.9333	-2.673 to 0.8059	No	ns	0.2861
8	0.6250	-1.114 to 2.364	No	ns	0.4736
9	1.558	-0.1809 to 3.298	No	ns	0.0779
10	0.1000	-1.639 to 1.839	No	ns	0.9085

2-10 6mm Balance Beam time pried – mix effects model (REML)) WT-VEH vs SAC1-VEH

Uncorrected Fisher's LSD

Week	Predicted (LS) mean diff.	95.00% CI of diff.	Below threshold?	Summary	Individual P Value
4	1.375	-1.993 to 4.743	No	ns	0.3665
5	1.907	0.3159 to 3.499	Yes	*	0.0253
6	1.190	-1.056 to 3.436	No	ns	0.2505
7	3.329	1.940 to 4.717	Yes	***	0.0008
8	3.273	0.6715 to 5.875	Yes	*	0.0207
9	4.398	2.564 to 6.232	Yes	***	0.0008
10	5.231	3.203 to 7.260	Yes	***	0.0005

2-10 6mm Balance Beam time pried – mix effects model (REML) SCA1-VEH vs SAC1-FINGO

Uncorrected Fisher's LSD

Week	Predicted (LS) mean diff.	95.00% CI of diff.	Below threshold?	Summary	Individual P Value
4	-0.008333	-3.583 to 3.566	No	ns	0.9958
5	-0.8667	-2.586 to 0.8527	No	ns	0.2721
6	0.4917	-2.100 to 3.083	No	ns	0.6673
7	1.042	-1.294 to 3.377	No	ns	0.3267
8	0.04167	-2.867 to 2.950	No	ns	0.9739
9	-0.8833	-3.705 to 1.938	No	ns	0.4832
10	-2.383	-4.217 to -0.5493	Yes	*	0.0180

2-10 6mm Balance Beam foot slips – mix effects model (REML) WT-VEH vs SAC1-VEH

Uncorrected Fisher's LSD

Week	Predicted (LS) mean diff.	95.00% CI of diff.	Below threshold?	Summary	Individual P Value
4	1.884	-1.256 to 5.025	No	ns	0.2336
5	3.472	0.3320 to 6.613	Yes	*	0.0309
6	2.706	-0.4344 to 5.846	No	ns	0.0896
7	5.288	2.148 to 8.429	Yes	**	0.0014
8	6.010	2.870 to 9.151	Yes	***	0.0003
9	7.200	4.059 to 10.34	Yes	***	<0.0001
10	7.957	4.817 to 11.10	Yes	***	<0.0001

2-10 6mm Balance Beam foot slips – mix effects model (REML) SCA1-VEH vs SAC1-FINGO

Uncorrected Fisher's LSD

Week	Predicted (LS) mean diff.	95.00% CI of diff.	Below threshold?	Summary	Individual P Value
4	-1.858	-6.815 to 3.099	No	ns	0.9169
5	-2.417	-7.374 to 2.540	No	ns	0.7476
6	-0.5333	-5.490 to 4.424	No	ns	>0.9999
7	-1.450	-6.407 to 3.507	No	ns	0.9771
8	-1.192	-6.149 to 3.765	No	ns	0.9926
9	-1.325	-6.282 to 3.632	No	ns	0.9862
10	-3.125	-8.082 to 1.832	No	ns	0.4582

5-13 Rotarod data – mix effects model(REML) WT-VEH vs SAC1-VEH

Uncorrected Fisher's LSD

Week	Predicted (LS) mean diff.	95.00% CI of diff.	Below threshold?	Summary	Individual P Value
5	-54.72	-94.25 to -15.19	Yes	*	0.0111
6	-79.03	-136.5 to -21.55	Yes	*	0.0115
7	-83.94	-138.5 to -29.38	Yes	**	0.0061
8	-76.58	-114.1 to -39.07	Yes	***	0.0009
9	-97.56	-136.5 to -58.59	Yes	***	0.0002
10	-106.8	-139.4 to -74.11	Yes	***	<0.0001
11	-90.25	-128.8 to -51.69	Yes	***	0.0003
12	-87.94	-134.9 to -40.97	Yes	**	0.0017
13	-85.72	-136.5 to -34.98	Yes	**	0.0034

5-13 Rotarod data – mix effects model (REML) SCA1-VEH vs SAC1-FINGO

Uncorrected Fisher's LSD

Week	Predicted (LS) mean diff.	95.00% CI of diff.	Below threshold?	Summary	Individual P Value
5	-3.194	-66.27 to 59.88	No	ns	0.9132
6	23.06	-38.47 to 84.58	No	ns	0.4270
7	2.167	-62.09 to 66.42	No	ns	0.9422
8	11.25	-40.38 to 62.88	No	ns	0.6409
9	32.42	-4.123 to 68.96	No	ns	0.0768
10	19.39	-42.59 to 81.37	No	ns	0.5054
11	16.06	-41.59 to 73.70	No	ns	0.5523
12	28.83	-33.82 to 91.49	No	ns	0.3329
13	13.39	-41.19 to 67.97	No	ns	0.6000

5-13 12mm Balance Beam time pried – mix effects model (REML)) WT-VEH vs SAC1-VEH

Uncorrected Fisher's LSD

Week	Predicted (LS) mean diff.	95.00% CI of diff.	Below threshold?	Summary	Individual P Value
5	3.472	0.09664 to 6.848	Yes	*	0.0439
6	1.194	-2.181 to 4.570	No	ns	0.4838
7	0.3611	-3.014 to 3.737	No	ns	0.8321
8	6.500	3.124 to 9.876	Yes	***	0.0002
9	5.333	1.958 to 8.709	Yes	**	0.0023
10	8.667	5.291 to 12.04	Yes	***	<0.0001
11	6.528	3.152 to 9.903	Yes	***	0.0002
12	6.250	2.874 to 9.626	Yes	***	0.0004
13	3.125	-0.2506 to 6.501	No	ns	0.0692

5-13 12mm Balance Beam time pried – mix effects model (REML) SCA1-VEH vs SAC1-FINGO

Uncorrected Fisher's LSD

Week	Predicted (LS) mean diff.	95.00% CI of diff.	Below threshold?	Summary	Individual P Value
5	2.861	-2.366 to 8.088	No	ns	0.2801
6	2.833	-2.394 to 8.060	No	ns	0.2847
7	3.139	-2.088 to 8.366	No	ns	0.2363
8	1.139	-4.088 to 6.366	No	ns	0.6664
9	4.083	-1.144 to 9.310	No	ns	0.1243
10	-2.472	-7.699 to 2.755	No	ns	0.3503
11	-1.389	-6.616 to 3.838	No	ns	0.5992
12	-2.917	-8.144 to 2.310	No	ns	0.2709
13	-3.778	-9.005 to 1.449	No	ns	0.1547

5-13 12mm Balance Beam foot slips – mix effects model (REML) WT-VEH vs SAC1-VEH

Uncorrected Fisher's LSD

Week	Predicted (LS) mean diff.	95.00% CI of diff.	Below threshold?	Summary	Individual P Value
5	0.3611	-1.217 to 1.939	No	ns	0.6508
6	0.2500	-1.328 to 1.828	No	ns	0.7539
7	1.417	-0.1614 to 2.995	No	ns	0.0779
8	2.111	0.5330 to 3.689	Yes	**	0.0093
9	1.222	-0.3558 to 2.800	No	ns	0.1275
10	3.667	2.089 to 5.245	Yes	***	<0.0001
11	2.444	0.8664 to 4.023	Yes	**	0.0027
12	4.083	2.505 to 5.661	Yes	***	<0.0001
13	2.139	0.5608 to 3.717	Yes	**	0.0084

5-13 12mm Balance Beam foot slips – mix effects model (REML) SCA1-VEH vs SAC1-FINGO

Uncorrected Fisher's LSD

Week	Predicted (LS) mean diff.	95.00% CI of diff.	Below threshold?	Summary	Individual P Value
5	0.9722	-0.6347 to 2.579	No	ns	0.2324
6	1.417	-0.1902 to 3.024	No	ns	0.0833
7	1.389	-0.2180 to 2.996	No	ns	0.0894
8	2.278	0.6709 to 3.885	Yes	**	0.0060
9	2.611	1.004 to 4.218	Yes	**	0.0017
10	0.2222	-1.385 to 1.829	No	ns	0.7841
11	-1.194	-2.801 to 0.4125	No	ns	0.1432
12	-2.306	-3.912 to -0.6987	Yes	**	0.0054
13	-1.056	-2.662 to 0.5513	No	ns	0.1952

5-13 6mm Balance Beam time pried – mix effects model (REML)) WT-VEH vs SAC1-VEH

Uncorrected Fisher's LSD

Week	Predicted (LS) mean diff.	95.00% CI of diff.	Below threshold?	Summary	Individual P Value
5	3.000	-2.035 to 8.035	No	ns	0.2164
6	4.583	-2.739 to 11.91	No	ns	0.1957
7	5.694	-1.110 to 12.50	No	ns	0.0926
8	7.889	3.615 to 12.16	Yes	**	0.0019
9	6.694	0.8283 to 12.56	Yes	*	0.0289
10	4.792	-0.4875 to 10.07	No	ns	0.0711
11	3.000	0.04045 to 5.960	Yes	*	0.0474
12	7.222	0.6370 to 13.81	Yes	*	0.0344
13	5.722	2.209 to 9.236	Yes	**	0.0043

5-13 6mm Balance Beam time pried – mix effects model (REML) SCA1-VEH vs SAC1-FINGO

Uncorrected Fisher's LSD

Week	Predicted (LS) mean diff.	95.00% CI of diff.	Below threshold?	Summary	Individual P Value
5	3.000	-2.035 to 8.035	No	ns	0.2164
6	4.583	-2.739 to 11.91	No	ns	0.1957
7	5.694	-1.110 to 12.50	No	ns	0.0926
8	7.889	3.615 to 12.16	Yes	**	0.0019
9	6.694	0.8283 to 12.56	Yes	*	0.0289
10	4.792	-0.4875 to 10.07	No	ns	0.0711
11	3.000	0.04045 to 5.960	Yes	*	0.0474
12	7.222	0.6370 to 13.81	Yes	*	0.0344
13	5.722	2.209 to 9.236	Yes	**	0.0043

5-13 6mm Balance Beam foot slips – mix effects model (REML) WT-VEH vs SAC1-VEH

Uncorrected Fisher's LSD

Week	Predicted (LS) mean diff.	95.00% CI of diff.	Below threshold?	Summary	Individual P Value
5	1.278	-0.2131 to 2.769	No	ns	0.0920
6	1.917	0.4258 to 3.408	Yes	*	0.0123
7	3.833	2.342 to 5.324	Yes	***	<0.0001
8	3.639	2.148 to 5.130	Yes	***	<0.0001
9	8.056	6.565 to 9.546	Yes	***	<0.0001
10	4.292	2.801 to 5.783	Yes	***	<0.0001
11	3.972	2.481 to 5.463	Yes	***	<0.0001
12	7.361	5.870 to 8.852	Yes	***	<0.0001
13	4.556	3.065 to 6.046	Yes	***	<0.0001

5-13 6mm Balance Beam foot slips – mix effects model (REML) SCA1-VEH vs SAC1-FINGO

Uncorrected Fisher's LSD

Week	Predicted (LS) mean diff.	95.00% CI of diff.	Below threshold?	Summary	Individual P Value
5	4.222	0.5698 to 7.875	Yes	*	0.0134
6	3.361	-0.2913 to 7.014	No	ns	0.0920
7	6.250	2.598 to 9.902	Yes	***	<0.0001
8	3.806	0.1531 to 7.458	Yes	*	0.0356
9	-1.611	-5.264 to 2.041	No	ns	0.8860
10	3.819	0.1670 to 7.472	Yes	*	0.0345
11	1.889	-1.764 to 5.541	No	ns	0.7588
12	-1.778	-5.430 to 1.875	No	ns	0.8153
13	0.7500	-2.902 to 4.402	No	ns	0.9994

10-13 Rotarod data – mix effects model(REML) WT-VEH vs SAC1-VEH

Uncorrected Fisher's LSD

Week	Predicted (LS) mean diff.	95.00% CI of diff.	Below threshold?	Summary	Individual P Value
10	-12.50	-44.72 to 19.72	No	ns	0.4356
11	-32.78	-65.00 to -0.5549	Yes	*	0.0464
12	-70.67	-102.9 to -38.44	Yes	***	<0.0001
13	-58.06	-90.28 to -25.83	Yes	***	0.0009

10-13 Rotarod data – mix effects model (REML) SCA1-VEH vs SAC1-FINGO

Uncorrected Fisher's LSD

Week	Predicted (LS) mean diff.	95.00% CI of diff.	Below threshold?	Summary	Individual P Value
10	-7.970	-48.00 to 32.06	No	ns	0.6902
11	1.083	-38.95 to 41.11	No	ns	0.9568
12	-0.1100	-40.14 to 39.92	No	ns	0.9956
13	-20.19	-60.22 to 19.83	No	ns	0.3148

10-13 12mm Balance Beam time pried – mix effects model (REML)) WT-VEH vs SAC1-VEH

Uncorrected Fisher's LSD

Week	Predicted (LS) mean diff.	95.00% CI of diff.	Below threshold?	Summary	Individual P Value
10	4.055	-0.5804 to 8.690	No	ns	0.0848
11	5.887	1.252 to 10.52	Yes	*	0.0140
12	2.805	-1.830 to 7.441	No	ns	0.2291
13	3.805	-0.8304 to 8.440	No	ns	0.1052

10-13 12mm Balance Beam time pried – mix effects model (REML) SCA1-VEH vs SAC1-FINGO

Uncorrected Fisher's LSD

Week	Predicted (LS) mean diff.	95.00% CI of diff.	Below threshold?	Summary	Individual P Value
10	-1.499	-4.994 to 1.995	No	ns	0.3889
11	-2.861	-6.355 to 0.6336	No	ns	0.1053
12	-1.500	-4.994 to 1.994	No	ns	0.3888
13	-1.861	-5.355 to 1.633	No	ns	0.2864

10-13 12mm Balance Beam foot slips – mix effects model (REML) WT-VEH vs SAC1-VEH

Uncorrected Fisher's LSD

Week	Predicted (LS) mean diff.	95.00% CI of diff.	Below threshold?	Summary	Individual P Value
10	4.334	3.283 to 5.385	Yes	***	<0.0001
11	3.835	2.784 to 4.885	Yes	***	<0.0001
12	3.472	2.421 to 4.523	Yes	***	<0.0001
13	3.417	2.366 to 4.468	Yes	***	<0.0001

10-13 12mm Balance Beam foot slips – mix effects model (REML) SCA1-VEH vs SAC1-FINGO

Uncorrected Fisher's LSD

Week	Predicted (LS) mean diff.	95.00% CI of diff.	Below threshold?	Summary	Individual P Value
10	4.334	3.283 to 5.385	Yes	***	<0.0001
11	3.835	2.784 to 4.885	Yes	***	<0.0001
12	3.472	2.421 to 4.523	Yes	***	<0.0001
13	3.417	2.366 to 4.468	Yes	***	<0.0001

10-13 6mm Balance Beam time pried – mix effects model (REML)) WT-VEH vs SAC1-VEH

Uncorrected Fisher's LSD

Week	Predicted (LS) mean diff.	95.00% CI of diff.	Below threshold?	Summary	Individual P Value
10	3.584	0.1230 to 7.044	Yes	*	0.0427
11	2.414	-1.047 to 5.875	No	ns	0.1667
12	3.554	0.09351 to 7.015	Yes	*	0.0444
13	2.817	-0.7102 to 6.343	No	ns	0.1146

10-13 6mm Balance Beam time pried – mix effects model (REML) SCA1-VEH vs SAC1-FINGO

Uncorrected Fisher's LSD

Week	Predicted (LS) mean diff.	95.00% CI of diff.	Below threshold?	Summary	Individual P Value
10	-2.249	-6.206 to 1.707	No	ns	0.2580
11	-2.582	-6.538 to 1.375	No	ns	0.1952
12	-1.721	-5.677 to 2.236	No	ns	0.3853
13	-2.994	-7.011 to 1.023	No	ns	0.1401

10-13 6mm Balance Beam foot slips – mix effects model (REML) WT-VEH vs SAC1-VEH

Uncorrected Fisher's LSD

Week	Predicted (LS) mean diff.	95.00% CI of diff.	Below threshold?	Summary	Individual P Value
10	4.028	2.394 to 5.661	Yes	***	<0.0001
11	5.917	4.283 to 7.550	Yes	***	<0.0001
12	4.639	3.005 to 6.272	Yes	***	<0.0001
13	5.136	3.470 to 6.802	Yes	***	<0.0001

10-13 6mm Balance Beam foot slips – mix effects model (REML) SCA1-VEH vs SAC1-FINGO

Uncorrected Fisher's LSD

Week	Predicted (LS) mean diff.	95.00% CI of diff.	Below threshold?	Summary	Individual P Value
10	-0.3317	-2.224 to 1.560	No	ns	0.7254
11	-2.333	-4.224 to -0.4405	Yes	*	0.0169
12	-1.889	-3.781 to 0.002791	No	ns	0.0503
13	-1.175	-3.101 to 0.7512	No	ns	0.2254
